# The Role of Pregnancy-Associated Hormones in the Development and Function of Regulatory B Cells

**DOI:** 10.3389/fendo.2014.00039

**Published:** 2014-04-01

**Authors:** Damián Muzzio, Marek Zygmunt, Federico Jensen

**Affiliations:** ^1^Research Laboratory, Department of Obstetrics and Gynecology, University of Greifswald, Greifswald, Germany

**Keywords:** regulatory B cells, pregnancy, female hormones, autoimmune diseases, estradiol, hCG

## Abstract

During mammalian pregnancy, highly specialized mechanisms of immune tolerance are triggered in order to allow the semi-allogeneic fetus to grow within the maternal uterus in harmony with the maternal immune system. Among other mechanisms, changes in the endocrine status have been proposed to be at least part of the machinery responsible for the induction of immune tolerance during pregnancy. Indeed, pregnancy-associated hormones, estradiol, progesterone, and human chorionic gonadotropin are known to confer immune suppressive capacity to innate as well as adaptive immune cells. Regulatory B cells, a subpopulation of B lymphocytes with strong immunosuppressive functions, were shown to expand during pregnancy. Furthermore, it is well-known that some women suffering from multiple sclerosis, significantly improve their symptoms during pregnancy and this was attributed to the effect of female sex hormones. Accordingly, estradiol protects mice from developing experimental autoimmune encephalomyelitis by triggering the expansion and activation of regulatory B cells. In this review, we discuss different mechanisms associated with the development, activation, and function of regulatory B cells with a special focus on those involving pregnancy-associated hormones.

## Introduction

Pregnancy in mammals is accompanied by profound changes and adaptations in the endocrine as well as the immune systems. These adaptations are necessary to allow the semi-allogeneic fetus to grow within the maternal uterus. However, in some cases, they may influence the course of pre-existing pathologies, as for the case of autoimmune diseases. In this regard, it is known that women suffering from a pre-existing autoimmune disease demonstrate changes in disease expression during pregnancy and this has been associated with changes in female sex hormones.

Early in pregnancy, just after conception has taken place, a systemic rise of progesterone (P4) and estradiol (E2) levels produced by corpora lutea (CL) in the ovary is evidenced. These hormones promote modifications of the uterine epithelium that allow a proper interaction with the blastocyst ensuring embryo implantation. Once the embryo is implanted, trophoblast cells produce and secrete increasing levels of human chorionic gonadotropin (hCG), which on the one side stimulates the CLs to continue producing progesterone, estradiol (E2), and estrone (E1) (1) and on the other side promotes angiogenesis in the uterine endothelium (2) as well as trophoblast migration and invasion into the uterine wall (3). Besides these widely accepted functions of female sex hormones in coordinating and controlling anatomical modifications associated with gravity, abundant evidence has also highlighted their role in shaping immune cells toward a transient state of tolerance necessary for the maintenance of pregnancy (4).

Indeed, a major challenge faced by the maternal immune system during pregnancy is precisely one of the most important immunological riddles. The mother’s immune system has to accept a semi-allogeneic fetus expressing foreign antigens through a state of immunological tolerance. At the same time, the mother and the fetus should be properly protected against the threat of infections. To achieve this, the immune machinery must be strictly regulated during the different stages of pregnancy. Several cell types with regulatory capacities have been described among T cells and B cells. Though the function of regulatory T cells (Tregs) in pregnancy has been extensively studied (5, 6), the role of regulatory B cells in this context is still a novel field to be explored.

## Regulatory B Cells and Their Emerging Role in the Context of Pregnancy

The first reports linking suppressive activities to B cells arose from experiments in guinea pigs, in which the transfer of B-cell-depleted splenocytes failed to inhibit delayed-type hypersensitivity (DTH) skin reactions (7, 8). More recently, strong evidence of the existence of B cells with suppressive capacities emerged in a murine model of multiple sclerosis (MS) called experimental autoimmune encephalomyelitis (EAE). In this model, mice lacking B cells suffer an exacerbated form of the disease (9). Later on, Fillatreau et al. showed similar results in EAE mice where the B cells are unable to produce the anti-inflammatory cytokine, IL-10 (10). From this point, the idea that B cells could be more than just antibody-producing cells and could regulate the immune responses in an IL-10-dependent fashion, started to be reinforced.

In both mouse and humans, different regulatory B-cell populations have been described. There is still controversy regarding their phenotype, but all of them show necessarily an *in vivo* suppressing capacity and their hallmark is the production of IL-10. In mice, Evans and co-authors have described a subset of Transitional T2 B cells with CD21^hi^CD23^+^ phenotype, able to synthesize IL-10 after stimulation of CD40 (11). Almost simultaneously, Yanaba et al. associated a CD5^+^CD1d^+^ B-cell population with the ability to produce IL-10 after stimulation with PMA and ionomycin, this population was named regulatory B10 cells (12). In humans, the phenotype is less clear. Blair and colleagues have found within the CD19^+^CD24^+^CD38^hi^ population, cells capable of suppressing, through IL-10 production, the differentiation of Th1 T cells after CD40 stimulation (13). Other authors confer the CD24^hi^CD27^+^ phenotype, which parallels the regulatory B10 cells in mouse, to B cells with the ability to inhibit pro-inflammatory responses via IL-10 secretion (14).

In the context of pregnancy, it was not until recently that a role for regulatory B cells has been introduced. In a murine model of pregnancy loss, Jensen et al. showed that CD19^+^CD5^+^CD1d^+^ regulatory B10 cells are diminished in the abortion-prone animals, when compared to those with normal pregnancies. Moreover, emulating the abortion preventing capacity of IL-10 administration (15), the transfer of IL-10-producing regulatory B10 cells was capable of reverting fetus rejection in otherwise aborting animals (16). The same group found that a population of CD19^+^CD24^hi^CD27^+^ regulatory B cells increases in the first trimester of pregnancy. Interestingly, the levels of these cells in patients suffering from spontaneous abortions remain as low as in non-pregnant women (17).

It is not yet clear why and how these changes in regulatory B cells during pregnancy are produced. The fact that B cells express receptors for hormones playing a fundamental role during pregnancy, especially hCG and E2, highlights the importance of further analyzing the interaction of these hormones with regulatory B cells.

## Female Sex Hormones as Modulators of Regulatory B Cells Activation and Function

As mentioned above, female sex hormones have long been suspected to influence immune responses. A strong clue for a role for female sex hormones in immunity is the fact that women are much more susceptible to developing autoimmune diseases than men (18, 19). In keeping with this, it is also well-documented that the symptoms of autoimmune diseases during pregnancy, when levels of female sex hormones are drastically modified, are significantly affected. In this regard, women suffering from autoimmune diseases depending on polyclonal B-cell activation and circulating immune complexes such as systemic lupus erythematosus (SLE) usually worsen during pregnancy (20, 21). However, other inflammation-driven conditions, such as rheumatoid arthritis (RA) or MS, are reported to ameliorate (22).

The hCG is a glycoprotein hormone synthesized by the syncytiotrophoblast immediately after embryo implantation. HCG secretion is mandatory for the maintenance of the E2 and P4 production by the corpus luteum during pregnancy until the placenta is developed and takes over the production of these hormones (23). Beside this fundamental role, in the past years, evidence has accumulated demonstrating an immunomodulatory capacity of hCG hormone on T cells (24–26), uNK cells (27), among others (28, 29). A recent work shifts the focus to the regulatory B cells (17).

It has been demonstrated that not only up to 95% of the CD19^+^CD24^hi^CD27^+^ regulatory B cells express the receptor for hCG (LH/hCGR) but that the addition of a human recombinant hCG to pure isolated CD19^+^ B cells *in vitro* induced a strong production of the potent anti-inflammatory and regulatory B-cell hallmark, IL-10 (17).

**Figure 1:**
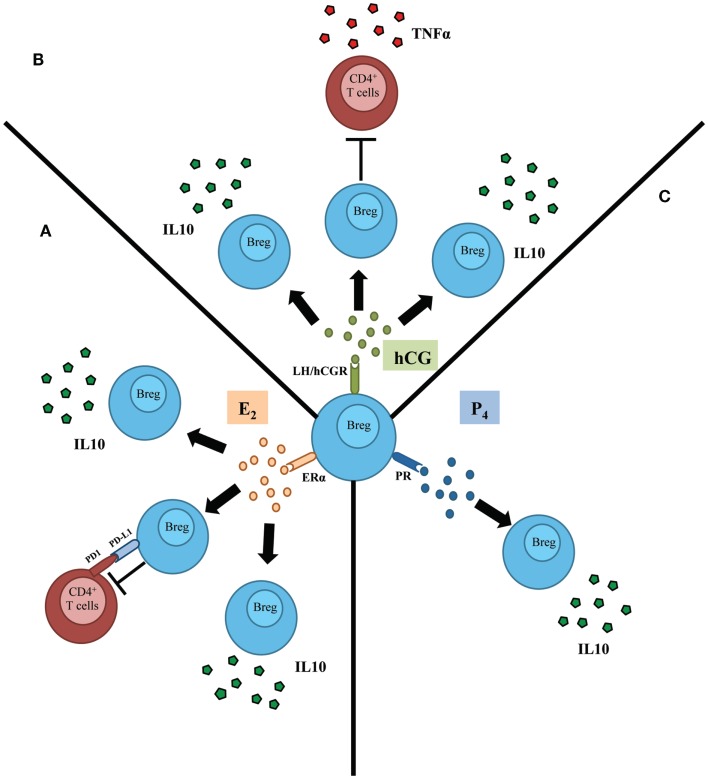
**Schematic representation depicting the influence of female sex hormones in the activation and subsequent function of regulatory B cells (Bregs)**. **(A)** Estradiol (E2), acting through estradiol receptor alpha (ERα), induces the proliferation as well as the production of IL-10 by regulatory B cells (Breg). In addition, E2 mediates the control of T cells’ activation by B cells throughout a mechanism involving PDL1/PD1 pathway. **(B)** Human chorionic gonadotropin (hCG) was proposed to induce proliferation of regulatory B cells. Besides, this hormone induces the production of IL-10 by B cells that in turn inhibit the production of TNFα by T cells. **(C)** Progesterone (P4) induces the production of the potent anti-inflammatory and Breg hallmark cytokine IL-10 by B cells.

Based on these results, the authors have proposed that hCG may drive an expansion of IL-10-producing regulatory B cells during normal pregnancy, thus controlling undesired immune activation that may compromise pregnancy well-being. Indeed, B cells from pregnant women undergoing normal pregnancies but not from patients suffering spontaneous pregnancy loss were able to suppress TNF-α production, a classic pro-inflammatory cytokine, by activated T cells (Figure 1) (17).

A very interesting phenomenon concerning the relationship between female sex hormones and regulatory B cells is the clinical remission that women suffering from MS experience during pregnancy (30). Subramanian et al. carried out a set of experiments that led to the conclusion that sex hormones have a positive effect on the activity of regulatory B cells that confer protection against the symptoms of the disease. In a recent manuscript, Subramanian and coworkers highlighted the role of regulatory B cells in the E2-mediated protection of EAE in mice lacking Tregs (31). In those mice, treatment with E2 provoked an augmentation of the production of IL-10 in splenocytes and up-regulation of the negative co-activation molecules, PDL1 and PDL2 on B cells. These results were in concordance with previous reports in which regulatory B cells conferred protection against EAE through production of IL-10 (10, 32, 33).

There are two receptors that mediate the actions of E2: ER-α and ER-β (34). It was first demonstrated that the protective effects of E2 in EAE are mediated by ER-α (35). It is known that B lymphocytes express ER-α and its activation affects the development, survival, expansion, and maturation of these cells (36–38). Recently, Bodhankar et al. showed that in the context of EAE, E2 acts in an ER-α-dependent way through B cells and not T cells. Notably, these protective effects of E2 are lost in the absence of B cells (39).

The scenario presented here centers on the regulatory B cells that, as a consequence of the higher levels of E2 in pregnancy, launch a set of suppressive mechanisms (IL-10 production, expression of PDL1) that favors immune tolerance (Figure 1). This probable natural necessity in pregnancy may be one reason for the clinical improvement observed in women with MS during gravity.

The steroid hormone progesterone is essential for the implantation as well as for the development of an adequate uterine structure, which involves changes in the growth and differentiation of the epithelial and stromal cell layers of the endometrial compartment that enables the maintenance of pregnancy (40). Several studies have established the importance of this hormone influencing the activity of various types of immune cells, such as the ability to inhibit the presenting capacity of dendritic cells (DCs) (41), monocytes, and macrophages (42, 43). Progesterone also has an impact on the levels of galectin-1, which in turn promotes the expansion and recruitment of suppressive uterine DCs (44).

An immunomodulatory action of P4 on B cells has also been described (45, 46). Treatment with P4 reduces the severity of EAE, elevating the levels of IL-10 and the numbers of CD19^+^ cells (47) (Figure 1). This work invites us to speculate, that as for hCG and E2, P4 could have an immunomodulatory action on regulatory B cells that may play a beneficial role in the remission of MS during gravity and the phenomenon of regulated tolerance in pregnancy.

## Conclusion

We describe in this review a B-cell subset capable of being activated and expanded upon hormone treatment, with the ability to suppress pro-inflammatory responses, leading to protection against EAE or pregnancy loss. Many therapeutic approaches for the treatment of autoimmune disease are centered on the depletion of B cells. Therefore, unspecific deletion of B cells as in the case of Rituximab™ treatment in MS patients (48) may result in the loss of a potentially valuable subset of B cells that mediate a beneficial anti-inflammatory environment associated with the transient remission of the disease. A better understanding of the mechanisms behind the activation and expansion of this population of regulatory B cells can provide novel therapeutic strategies against autoimmune diseases like MS as well as to pregnancy-associated disturbances.

## Conflict of Interest Statement

The authors declare that the research was conducted in the absence of any commercial or financial relationships that could be construed as a potential conflict of interest.
